# Assessment of Psychosocial Functioning of Mothers of Children with Diabetes Mellitus Compared to Mothers of Healthy Children

**DOI:** 10.1155/2019/6821575

**Published:** 2019-04-09

**Authors:** Marta Makara-Studzińska, Siva Somasundaram, Ghulam Md. Ashraf, Małgorzata Gogacz, Agata Madej, Bernadetta Izydorczyk, Jerzy Leszek, Svetlana A. Lebedeva, Vladimir N. Chubarev, Vadim V. Tarasov, Eric Kirkland, Gjumrakch Aliev

**Affiliations:** ^1^Health Psychology Unit, Collegium Medicum, Jagiellonian University, Kraków, Poland; ^2^Department of Biological Sciences, Salem University, Salem, WV 26426, USA; ^3^King Fahd Medical Research Center, King Abdulaziz University, Jeddah, Saudi Arabia; ^4^Department of Applied Psychology, Medical University of Lublin, Poland; ^5^Institute of Applied Psychology, Jagiellonian University, Kraków, Poland; ^6^Department of Psychiatry, Wroclaw Medical University, Wroclaw, Poland; ^7^Sechenov First Moscow State Medical University (Sechenov University), 119991, Moscow, Russia; ^8^School of Education & Business, Professor and Vice President, Salem University, WV, 26426, USA; ^9^Institute of Physiologically Active Compounds, Russian Academy of Sciences, Chernogolovka, 142432, Russia; ^10^GALLY International Biomedical Research Institute, 7733 Louis Pasteur Drive, #330, San Antonio, TX, 78229, USA

## Abstract

Diabetes mellitus (DM) is a chronic disease requiring changes in the behaviour of the entire family. The responsibility for implementing doctor's recommendations falls mainly upon the mother. The aim of this study is to assess the psychosocial functioning of mothers of children with DM compared to mothers of healthy children. The study involved 120 mothers: 60 with children with DM and 60 with healthy children. Data were collected using an original social-demographic questionnaire developed by the authors as well as Antonovsky's Sense of Coherence Scale (SOC-29), Schwarzer and Schultz's Berlin Social Support Scales (BSSS), Rosenberg's Self-Esteem Scale (SES), and Zigmond and Snaith's Hospital Anxiety and Depression Scale (HADS). The assessment scales were standardised and accredited by the Polish Psychological Association. The results suggest that DM in children has no effect on the psychosocial functioning of mothers regarding their self-esteem and sense of coherence. However, mothers of children with DM are well-prepared for living in a difficult situation. Social support offered to mothers of diabetic children helps them to maintain their psychosocial health.

## 1. Introduction

It has been reported that about 1400 children in Poland were diagnosed with diabetes mellitus (DM) in 2012[1]. DM is a chronic metabolic disorder characterised by deficiency of insulin or insulin action leading to hyperglycaemia. The causative factors include genetic disorders and influence of the external environment, which can cause an insufficient insulin level or insulin resistance [2]. The World Health Organization (WHO) distinguished two classifications of DM.

- Type 1 (T1DM) is insulin-dependent and commonly first diagnosed in childhood or puberty.
- Type 2 (T2DM) is insulin-independent and mainly affects individuals over 30 years of age [2].

In the past, DM was classified as one of the greatest global health issues. The United Nations considers it a disease of civilization. It was classified as one of four globally relevant noncommunicable diseases that most afflict mankind worldwide [3, 4]. A child suffering from T1DM requires long-term care that entails considerable on-going effort and sacrifice on the part of the entire family [5–7]. Diabetologists recommend that effective treatment of DM depends on effective education of caregivers to implement the comprehensive treatment that is necessary to maintain the child's health. This care involves regular attention by medical specialists, nurses, psychologists, and dietitians [8]. Mothers play a centrally important role in managing the health care of diabetic children [9–11].

To be successful, mothers need to adapt to the needs of their children for holistic care. As but one example, many children with DM suffer from sleep disorders due to their strong emotions surrounding the disease and its progression [9–11]. Lustman* et al.* established that daily health-maintenance activity related to the disease constitute a burden for both the mother and the child [12]. Some recommend the parents, especially mothers, need to adopt a specific form of nurturing mother-child relationship.

According to Wojciechowska* et al.,* parents' involvement in the treatment should focus on engagement and care within the family unit as a collaboration of partners [13]. The mother must set specific goals for herself and appropriate tasks to be performed by the child (e.g., learning how to do blood testing for glucose levels on their own, developing health-promoting habits, and preventing complications). The mother also must monitor and evaluate her own and her child's compliance with treatment plans and achievement of specific goals.

DM also affects the perceived quality of life across the spectrum of mental, physical, and social areas of life and development. The concept of quality of life involves the child's health, physical functioning, well-being, satisfaction with life, and psychosocial adaptation [14].

The specific aim of this study is to assess the psychosocial functioning of mothers of diabetic children, compared to mothers of healthy children. The study involved 120 female participants, 60 of whom were taking care of children with diabetes compared to 60 with healthy children.

## 2. Materials and Methods

### 2.1. Study Group and Research Procedure

This project was approved by the Bioethical Committee of the Medical Institute in Lublin (Poland) no. KE-0254/123/2013. The study involved 120 female participants. A study of 60 mothers of diabetic children was conducted by the Professor Niewiedzioł's Support Group for Children and Adolescents with DM in Lublin between the years 2013 and 2015. The study of 60 mothers of healthy children was conducted in a nursery and a school.

The experimental group comprised mothers of diabetic children; the control group comprised mothers of healthy children. Respondents were informed of the research objective and were promised anonymity. They were assured that the findings would be used solely for scientific purposes. All participants completed informed consent forms.

### 2.2. Research Tools

Data were collected using an original social-demographic questionnaire developed by the authors as well as Antonovsky's Sense of Coherence Scale (SOC-29), Schwarzer and Schultz's Berlin Social Support Scales (BSSS), Rosenberg's Self-Esteem Scale (SES), and Zigmond and Snaith's Hospital Anxiety and Depression Scale (HADS). The assessment scales were standardised and accredited by the Polish Psychological Association.

#### 2.2.1. Original Social-Demographic Questionnaire

The questionnaires were completed individually in private. Demographic questions included the participant's age, place of residence, education, marital status, and source of income, as well as the sex and age of the child with T1DM (Tables 1(f) and 1(g)). Other questions pertained to problems encountered placing the child in day care, nursery, or school; the impact of the disease on learning; disease duration; alarming symptoms noticed before the diagnosis; the necessary care for the child as well as whether the child has and uses an insulin pump or the reasons for not having and using one. Some questions pertained to the mother's reflections on changes in her circumstances and behaviour after the child was diagnosed with DM. For example, the change of material conditions (insulin is expensive), the amount of time spent with the family, changes in relations with the partner since the child's diagnosis, and the group of people supporting the mother.

The remaining group of questions pertained to the child's ability to care for him- or herself (i.e., the ability to test blood sugar levels, the way insulin in administered, and whether dietary rules are observed). The results in the authors' original sociodemographic questionnaire enabled us to characterise the mothers participating in the study

The instrument is as follows (Tables 1(a)–1(g)). After reading the questionnaire, please kindly mark the selected answers with a cross and enter your own in the designated places.

#### 2.2.2. Antonovsky's Sense of Coherence Scale (SOC-29)

The SOC-29 measures an individual's general sense of coherence. The sense of coherence is operationally defined as feelings of being understood, resourceful, and sensible. The instrument has 29 items in the form of questions scored on a Likert-type scale from 1 to 7. The total score is the sum of a given individual's scores for the 29 items. The maximum possible score is 203, while the lowest is 29. Higher scores correlate positively with a stronger sense of coherence [15].

#### 2.2.3. Schwarzer & Schultz's Berlin Social Support Scales (BSSS)

BSSS is a set of scales used for measuring cognitive and behavioural dimensions of social support with special reference to individuals supporting the mothers of diabetic and healthy children. The scales were adapted to Polish by Łuszczyńska* et al*. [16]. The BSSS contains five subscales: (1) perceived available support, (2) need for support, (3) support seeking, (4) actually received support, and (5) protective buffering support. Protective buffering support is operationally defined as an amalgam of hiding worries, denying concerns, and yielding to one's partner in an effort to avoid disagreements and reduce one's partner's upset and burden. The answers were rated on a Likert-type scale from 1 to 4.

#### 2.2.4. Rosenberg's Self-Esteem Scale (SES)

The scale measures general self-esteem, operationally defined as a composite attitude towards oneself. The SES consists of 10 statements that respondents complete on a Likert-type scale from 1 to 4. The results can range from 10 to 40. The Polish adaptation of this method was developed by M. Łaguna, K. Lachowicz-Tabaczek, and I. Dzwonkowska [17].

#### 2.2.5. Zigmond and Snaith's Hospital Anxiety and Depression Scale (HADS)

It is used as a screening tool to identify anxiety and depressive symptoms and consists of two independent subscales. Each of them contains 7 statements on the actual condition of the participant. In total, the scale consists of 14 questions. The respondent assesses intensity of a given trait on a scale ranging from 0 to 3. The participants were subjected to the assessment only in the last week prior to the study. From among the above-described research tools, SOC-29, BSSS, SES, and HADS were accredited by the Polish Psychological Association.

### 2.3. Statistical Methods

The statistical analysis of the obtained results was conducted using IBM SPSS Statistics software. The following methods of statistical tests were used:

- Kolmogorov-Smirnov test
- Mann-Whitney U test
- Kruskal-Wallis test
- Spearman's rank order correlation

## 3. Results

Table 1 summarizes the original social-demographic questionnaire. It lists the results of diabetic children and healthy children and their mothers side by side.

The youngest mother of a diabetic child was 29 years, whereas the oldest was 60. The youngest mother of a healthy child was 27 and the oldest was 56. Thirty-four (56.7%) mothers of diabetic children resided in a city with a population over 100 thousand, while the group of mothers of healthy children had 29 (48.3%) who resided in towns with a population of 25-100 thousand.

The highest number of mothers of diabetic children (34 or 56.7%) and healthy children (42 or 70.0%) reported having completed higher education. Among the mothers of diabetic children, 50 (83.3%) and 54 (90%) of the mothers of healthy children were married. An analysis of the source of income showed that most of the study participants were professionally active in both the group of mothers of diabetic children (36 or 60%) and healthy children (52 or 86.7%).

The age of diabetic children ranged from 7 to 16 years. Twenty percent of them were 13 years old. Girls constituted 65% of the group, while boys accounted for 35%. Most often, the number of children in families of diabetic children was estimated at two (46.7%). Families with three children were the fewest (15%). During the study, a clear majority of diabetic children (56.7%) attended primary schools. A slightly lower percentage (40%) attended lower secondary schools, while the fewest children (3.3%) attended nurseries. Fifty-four (90%) of the surveyed mothers did not report any problems with finding a place for their children in day care/nursery/school. Based on the analysis, it was found that 73.3% of diabetic children did not notice any changes in learning, while 20% reported that learning deteriorated. About 6.7% of mothers reported their children to be more eager to study. Diabetic children were most often the second child in the family (40%) and somewhat less often the only child (35%) or the first child (20%). Most of the children suffered from the disease starting from 1.5 to 13 years of age. The mean age at the diagnosis was slightly over 5 years. Over half of the study participants (60%) noticed symptoms of the disease before the diagnosis, and 68.3% of the mothers declared that their children required continuous care.

A clear majority of the diabetic children (86.7%) had an insulin pump. The main cause for not having an insulin pump was the child's reluctance (55.6%), followed by fear (33.3%), and lack of information on the therapeutically insulin pump (11.1%).

Seventy-five percent (75%) of the mothers of diabetic children stated that their children could calculate carbohydrate exchanges. Most of the diabetic children (95%) could measure blood sugar levels by themselves. Half of the participants reported their children measured their blood sugar levels more than 5 times per day; 28.3% reported that it is done before every meal. According to 61.7% of mothers of diabetic children, their children followed the dietary rules and 28.3% of them complied with such recommendations shortly after the diagnosis, while the remaining 10% were not following such rules and never did. According to most of the respondents (60%), their financial conditions did not change from the time of diagnosis. The very same fraction (60%) of mothers spend the same amount of time with their families as before the diagnosis of the disease, and 79.7% of the participants reported that their relations with their partner did not change. However, the remaining 20.3% of the respondents indicated there was a change. Thirty-nine mothers (65%) of diabetic children received support from the immediate family, 28 (46.7%) from partners, 15 (25%) from friends, 10 (16.7%) from parents, and 4 (6.7%) from neighbours.

The group of healthy children consisted of 51 (51%) boys and 49 (49%) girls. Most children in this group were aged between 14 and 16 (13.6% each). Forty-one (44.6%) of them attended a lower secondary school, 40 (43.4%) attended primary school, and 11 (12%) attended a nursery. The majority of mothers (88.1%) of healthy children did not report any problems with finding a place for their children in a day care/nursery/school.

The results of the analyses presented in the tables indicate that both the mothers of diabetic children and the mothers of healthy children are not significantly different to each other regarding the level of self-esteem, sense of coherence, anxiety, and depression.

The mothers of diabetic children received most support from their immediate family (65%), followed by their husbands/partners (46.7%). The mothers of healthy children received similar perceived levels of support both from the immediate family and from husband/partner (70% each) (Table 2).

Statistically significant positive correlations were found between self-esteem levels and sense of coherence (Table 3). Therefore, the higher the self-esteem levels, the greater the sense of coherence in general and in its individual dimensions. This finding applied to both the mothers of diabetic children and the mothers of healthy children,

In both groups, statistically significant negative correlations were found between anxiety, depression, and sense of coherence (Table 4). Higher levels of anxiety and depression were correlated with the lower sense of coherence in general and in its individual dimensions. Statistically significant negative correlations were noted in the groups of mothers of diabetic children and healthy children between anxiety and depression and self-esteem levels. The higher the level of anxiety and depression, the lower the self-esteem levels in the study participants (Tables 4 and 5).

Only depression and support are correlated in a statistically significant manner (Table 6). Anxiety did not correlate significantly with support. In the group of mothers of diabetic children, high levels of protective buffering support were associated with the higher levels of depression. In contrast, in the group of mothers of healthy children, the higher the level of the dimensions of support (perceived general available support, perceived available emotional support, perceived available instrumental support, actually received general support, actually received emotional support, actually received institutional support, actually received informative support, satisfaction with actually received support), the lower the level of depression.

Positive correlations were found both in the group of mothers of diabetic children with the severity of DM as a cofounding factor and in the group of mothers of healthy children between self-esteem levels and support (Table 7). Among the mothers of diabetic children, the higher the level of specific dimensions of support (i.e., perceived general available support, perceived available emotional support, perceived available instrumental support, actually received general support, actually received emotional support, and actually received institutional support), the higher self-esteem levels. While among the mothers of healthy children, the higher the level of specific dimensions of support (perceived general available support, perceived available emotional support, perceived available instrumental support, actually received general support, actually received emotional support, actually received institutional support, and actually received informative support), the higher the self-esteem levels.

Statistically significant positive correlations were found among the mothers of diabetic children between senses of coherence and support (Table 8). The higher the perceived general available support, perceived available emotional support, and perceived available instrumental support, the higher the sense of coherence in general and in individual dimensions. The higher the actually received general support and actually received emotional support, the greater the sense of coherence in general and in all of its individual dimensions. The higher the actually received institutional support, the greater the sense of coherence in general and in all of its individual dimensions except for sensibleness. The higher the actually received informative support, the greater the sense of coherence in terms of resourcefulness. The greater the satisfaction with the actually received support, the greater the sense of being understood, sensibleness, and the general sense of coherence.

Statistically significant positive correlations occurred in the group of mothers of healthy children between the sense of coherence and support (Table 9). The higher the perceived general available support, perceived available emotional support, perceived available instrumental support, and actually received informative support, the greater sense of coherence in general and in all of its individual dimensions. In turn, the greater the actually received general support, actually received emotional support, actually received institutional support and satisfaction with actually received support, the greater sense of coherence in general and in all of its individual dimensions except for the sense of being understood.

## 4. Discussion

The aim of the study was to assess the psychosocial functioning of mothers of diabetic children compared to a control group of mothers of healthy children. Diabetic children receive care from their mothers at various ages. The mothers of diabetic children were aged from 29 to 60 years and the highest percentage of them resided in cities with a population over 100 thousand, while the lowest percentage lived in towns of up to 25 thousand. The research conducted by Gawłowicz [18] showed that 55% of the participants lived in rural areas, while 45% lived in urban areas. The mean age of mothers was estimated at around 40 years. Irrespective of the patients geographical locations the psychosocial functioning of mothers do not affect the diabetic care of their children.

The general sense of coherence and its constituent parts (sense of being understood, resourcefulness, and sensibleness) were subjected to analysis. The respondents obtained scores similar to the average. Studies conducted by Kurowska* et al*. [19] on the relation between sense of coherence and general mental health proved that in a group of people with a high level of sense of coherence symptoms of anxiety, insomnia, and depression are less common compared to individuals with a low level of sense of coherence (SOC). In turn, J. Dymecka [20] claims that a serious disease affecting a family member constitutes a source of great stress to a given family; hence, it is important to analyze sense of coherence in the mother-father system. Parents of children who experience a disability such as a chronic disease have a significantly lower sense of coherence than other parents [20]. In the source literature, a difference in a sense of coherence is reported between parents who accepted their child's disease and parents who cannot come to terms with the diagnosis [21]. Though, sense of coherence is a permanent parameter and the earlier findings suggested that the significant changes were found in patients with a low SOC level and the increase of SOC was observed after a 10-week treatment and was stable even after six months. In addition, the results suggest that the therapy can be more effective in patients with a low SOC level and not in high SOC [22].

The studies reported by Horsch* et al*. [23] showed that the mothers of diabetic children manifest symptoms typical of the posttraumatic stress disorders after the diagnosis. Seventeen percent (17%) of female participants report severe or moderate depressive symptoms, while 40% reported moderate or severe anxiety (Tables 4 and 5). According to Bowers* et al.* [24], a long-term sadness is reported by parents after their child was diagnosed with a chronic disease. Studies conducted by Kędziora [25] showed that mothers respond with an initial increase in anxiety that subsides to an extent after a year. A report on anxiety of mothers of children with DM and those with epilepsy manifest a similar average level of anxiety [26].

A mother caring for a child with a chronic disease begins a new period in her life that requires her to learn new skills, to set new achievable goals, and to deal effectively with her fears. After the diagnosis, mothers of diabetic children undertake new actions, overcoming their fears, and establishing new contacts with individuals who offer them support [13]. A positive role can be played by the family by actively engaging issues experienced by the diabetic child and by sharing tasks between all family members.

As a chronic disease, diabetes mellitus causes can be overcome using social support. This finding is similar to reports by the authors regarding adaptation to an incurable disease [27, 28]. Social support fulfills a significant role in the shaping of an individual's physical and mental condition. It prevents formation of adverse tension in contrast to situations where no support is provided to such a person [29, 30]. Pisula and Czaplińska [31] reported that the level of education of mothers of adolescents suffering from chronic diseases is positively correlated with the manner in which they cope with associated stress. The findings showed that received support has a positive effect on self-esteem levels. In the source literature, no studies were found that would directly pertain to the correlation between support and self-esteem levels in mothers caring for children with diabetes mellitus.

The results presented in this paper indicate that both the patient and the family require ongoing psychological assistance. Furthermore, the best results can be obtained through combining medical care and psychosocial support (Tables 6–9). An important part is played by psychoeducation, participation in support groups, and training sessions dedicated to the subject of coping with diabetes mellitus. The aggregate effects facilitate emotional adaptation to living with the disease for both the child and the family members.

## 5. Conclusions

Mothers of children diagnosed with diabetes mellitus are immersed suddenly in a difficult situation related to this chronic, progressive disease that has the potentially for serious, life-threatening effects on their children. Mothers of diabetic children who reported high self-esteem and general sense of coherence also reported were positively correlated with receiving professional counselling support that addresses specific issues and offers general information on additional sources of available help.

A high sense of coherence is reported as a health-promoting factor that facilitates choosing optimal behaviours that enable preserving good health, including mental health. Mothers of diabetic children and mothers of healthy children are not significantly different with respect to self-esteem levels, sense of coherence, anxiety, and depression. Positive correlations are enhanced by receiving professional medical and psychological help (e.g., the Support Group for Children and Adolescents with Diabetes), participation in training sessions, and support from loved ones.

## Figures and Tables

**Table:** (a) The age Factor of Mothers

*The age Factor*	N	Min	Max	Median	Mean	SD
The mothers of diabetic children	60	29	60	40	40.73	7.24
The mothers with healthy children	60	27	56	41	41.63	6.65

**Table:** (b) The Place of Residence

*Place of residence*	*Participants*			
	The mothers of diabetic children*∗*		The mothers with healthy children*∗*	
	N	%	N	%
Village	8	13.3	14	23.3
City up to 25 thousand inhabitants	6	10.0	8	13.3
City 25-100 thousand inhabitants	12	20.0	29	48.3
City over 100 thousand inhabitants	34	56.7	9	15.0

Total	60	100.0	60	100.0

*∗*Chi Sq. P < 0.001

**Table:** (c) Education of Mothers

*Education*	*Participants*			
	The mothers of diabetic children*∗*		The mothers with healthy children*∗*	
	N	%	N	%
Primary education	0	0.0	1	1.7
Vocational education	3	5.0	5	8.3
Secondary education	22	36.7	12	20.0
Higher education	34	56.7	42	70.0
Other	1	1.7	0	0.0

Total	60	100.0	60	100.0

*∗*Chi Sq. P < 0.2

**Table:** (d) The Marital status of Mothers

*Marital status*	*Participants*			
	The mothers of diabetic children*∗*		The mothers with healthy children*∗*	
	N	%	N	%
Unmarried	0	0.0	2	3.3
Married	50	83.3	54	90.0
Widow	2	3.3	0	0.0
Divorced	6	10.0	4	6.7
Free relationship	2	3.3	0	0.0

Total	60	100.0	60	100.0

*∗*Chi Sq. P < 0.2

**Table:** (e) The source of income of Mothers

*The source of income*	*Participants*			
	The mothers of diabetic children		The mothers of healthy children	
	N	%	N	%
Economic activity	11	18.3	5	8.3
Professional work	36	60.0	52	86.7
Pension	5	8.3	0	0.0
Old-age pension	3	5.0	0	0.0
Unemployed	5	8.3	3	5.0

Total	60	100.0	60	100.0

*∗*Chi Sq. P < 0.02

**Table:** (f) The Gender of Children

*Gender of Children*	*Participants*			
	diabetic children*∗*		healthy children*∗*	
	N	%	N	%
Female	65	65.0	49	49.0
Male	35	35.0	51	51.0

Total	100	100.0	100	100.0

*∗*Chi Sq. P < 0.05

**Table:** (g) The Status of Educating Schools

*Status of Educating Schools*	*Children*	
	Diabetic*∗*	Healthy*∗*
	%	%
Nursery	0.0	0.0
Preschool	3.3	12.0
Primary school	56.7	43.4
Middle school	40.0	44.6

Total	100.0	100.0

*∗*Chi Sq. P < 0.05

**Table:** (h) Hardship in finding nursery/preschool/school for child

*Hardship in finding nursery/preschool/school for child*	*Participants*			
	The mothers of diabetic children*∗*		The mothers with healthy children*∗*	
	N	%	N	%
Yes	6	10.0	7	11.9
No	54	90.0	52	88.1

Total	60	100.0	59	100.0

*∗*Chi Sq. P < 1.0

**Table 2:** Individuals supporting the mothers of diabetic and healthy children.

Who provides you with reliable support in difficult situations?	Group			
	mothers of diabetic children		mothers of healthy children	
	N	%*∗*	N	%*∗*
husband/partner	28	46.7%	42	70%
immediate family	39	65%	42	70%
neighbours	4	6.7%	10	16.7%
friends	15	25%	19	31.7%
parents of diabetic children	10	16.7%	0	0%

*∗*The total % is not equal to 100, as the participants were free to check any number of answers.

**Table 3:** Correlations between self-esteem and sense of coherence in the surveyed groups of women.

Sense of Coherence Scale (SOC-29)	Group			
	mothers of diabetic children		mothers of healthy children	
	Self-Esteem Scale		Self-Esteem Scale	
	rho	p	rho	p
Sense of being understood	0.562	<0. 001*∗∗*	0.458	<0. 001*∗∗*
Resourcefulness	0.389	0.002*∗∗*	0.475	<0. 001*∗∗*
Sensibleness	0.464	<0. 001*∗∗*	0.499	<0. 001*∗∗*
General sense of coherence	0.519	<0. 001*∗∗*	0.529	<0. 001*∗∗*

**Table 4:** Correlations between anxiety, depression, and sense of coherence in the groups of women.

Sense of Coherence Scale (SOC-29)	Group							
	mothers of diabetic children				mothers of healthy children			
	Anxiety		Depression		Anxiety		Depression	
	rho	P	rho	p	rho	p	rho	p
Sense of being understood	-0.522	<0.001*∗∗*	-0.408	0.001*∗∗*	-0.564	<0.001*∗∗*	-0.611	<0.001*∗∗*
Resourcefulness	-0.496	<0. 001*∗∗*	-0.479	<0.001*∗∗*	-0.599	<0.001*∗∗*	-0.649	<0. 001*∗∗*
Sensibleness	-0.469	<0. 001*∗∗*	-0.470	<0.001*∗∗*	-0.588	<0.001*∗∗*	-0.654	<0. 001*∗∗*
General sense of coherence	-0.559	<0. 001*∗∗*	-0.489	<0.001*∗∗*	-0.658	<0.001*∗∗*	-0.718	<0. 001*∗∗*

**Table 5:** Correlations between anxiety, depression, and level of self-esteem in the female participants.

	Self-Esteem Scale			
	Group			
	mothers of diabetic children		mothers of healthy children	
	Rho	p	rho	P
Anxiety	-0.481	<0. 001*∗∗*	-0.431	0.001*∗∗*
Depression	-0.476	<0. 001*∗∗*	-0.586	<0. 001*∗∗*

**Table 6:** Correlations between anxiety, depression, and support.

Berlin Social Support Scales (BSSS)	Group							
	mothers of diabetic children				mothers of healthy children			
	Anxiety		Depression		Anxiety		Depression	
	Rho	p	rho	P	rho	P	rho	p
Perceived general available support	-0.107	0.415	-0.193	0.139	-0.238	0.068	-0.422	0.001*∗∗*
Perceived available emotional support	-0.119	0.367	-0.177	0.177	-0.219	0.092	-0.339	0.008*∗∗*
Perceived available instrumental support	-0.065	0.622	-0.150	0.252	-0.226	0.083	-0.450	<0.001*∗∗*
Need for support	0.090	0.492	0.051	0.696	0.091	0.488	-0.103	0.433
Seeking support	-0.030	0.819	-0.104	0.429	-0.067	0.611	-0.187	0.152
Actually received general support	-0.223	0.087	-0.152	0.246	-0.180	0.169	-0.473	<0.001*∗∗*
Actually received emotional support	-0.137	0.295	-0.168	0.199	-0.145	0.268	-0.397	<0.002*∗∗*
Actually received institutional support	-0.165	0.208	-0.129	0.326	-0.075	0.566	-0.432	<0.001*∗∗*
Actually received informative support	-0.116	0.379	-0.035	0.790	-0.239	0.066	-0.338	<0.008*∗∗*
Satisfaction with actually received support	-0.119	0.367	-0.131	0.318	-0.081	0.539	-0.307	0.017*∗*
Protective buffering support	0.097	0.462	0.320	0.013*∗*	-0.078	0.553	-0.013	0.924

**Table 7:** Correlations between self-esteem and support.

Berlin Social Support Scales (BSSS)	Self-Esteem Scale			
	Group			
	mothers of diabetic children		mothers of healthy children	
	rho	P	Rho	P
Perceived general available support	0.394	0.002*∗∗*	0.582	<0. 001*∗∗*
Perceived available emotional support	0.396	0.002*∗∗*	0.526	<0. 001*∗∗*
Perceived available instrumental support	0.328	0.010*∗*	0.571	<0. 001*∗∗*
Need for support	0.005	0.969	0.118	0.369
Seeking support	0.231	0.075	0.180	0.168
Actually received general support	0.300	0.020*∗*	0.340	0.008*∗∗*
Actually received emotional support	0.330	0.010*∗*	0.349	0.006*∗∗*
Actually received institutional support	0.291	0.024*∗*	0.283	0.028*∗*
Actually received informative support	0.191	0.144	0.312	0.015*∗*
Satisfaction with actually received support	0.233	0.073	0.230	0.077
Protective buffering support	-0.072	0.583	-0.207	0.113

**Table 8:** Correlations between sense of coherence and support among the mothers of diabetic children.

Mothers of diabetic children	Sense of being understood		Resourcefulness		Sensibleness		General sense of coherence	
	rho	p	rho	P	rho	P	rho	P
Perceived general available support	0.408	<0.001*∗∗*	0.487	<0.001*∗∗*	0.405	0.001*∗∗*	0.463	<0. 001*∗∗*
Perceived available emotional support	0.450	<0.001*∗∗*	0.467	<0.001*∗∗*	0.348	0.006*∗∗*	0.457	<0. 001*∗∗*
Perceived available instrumental support	0.324	0.012*∗*	0.453	<0.001*∗∗*	0.400	0.002*∗∗*	0.418	<0.001*∗∗*
Need for support	0.031	0.813	0.110	0.403	0.079	0.548	0.074	0.574
Seeking support	0.240	0.065	0.181	0.167	0.143	0.275	0.206	0.114
Actually received general support	0.358	0.005*∗∗*	0.455	<0.001*∗∗*	0.285	0.027*∗*	0.414	0.001*∗∗*
Actually received emotional support	0.305	0.018*∗*	0.425	<0.001*∗∗*	0.307	0.017*∗*	0.395	0.002*∗∗*
Actually received institutional support	0.344	0.007*∗∗*	0.376	0.003*∗∗*	0.239	0.066	0.341	0.008*∗∗*
Actually received informative support	0.234	0.071	0.292	0.024*∗*	0.141	0.281	0.254	0.050
Satisfaction with actually received support	0.415	0.001*∗∗*	0.384	0.002*∗∗*	0.253	0.051	0.410	0.001*∗∗*
Protective buffering support	-0.044	0.737	-0.153	0.244	-0.089	0.497	-0.079	0.549

**Table 9:** Correlations between sense of coherence and support in groups of mothers of diabetic children.

Mothers of healthy children	Sense of being understood		Resourcefulness		Sensibleness		General sense of coherence	
	rho	p	rho	P	rho	P	rho	p
Perceived available general support	0.343	0.007*∗∗*	0.434	<0.001*∗∗*	0.377	0.003*∗∗*	0.418	0.001*∗∗*
Perceived available emotional support	0.334	0.009*∗∗*	0.396	0.002*∗∗*	0.299	0.020*∗*	0.377	0.003*∗∗*
Perceived available instrumental support	0.330	0.010*∗*	0.454	<0.001*∗∗*	0.424	0.001*∗∗*	0.438	<0. 001*∗∗*
Need for support	-0.116	0.378	-0.063	0.632	-0.092	0.485	-0.114	0.387
Support seeking	-0.100	0.448	0.007	0.959	-0.050	0.707	-0.053	0.689
Actually received general support	0.249	0.055	0.447	<0. 001*∗∗*	0.452	0.001*∗∗*	0.427	0.001*∗∗*
Actually received emotional support	0.195	0.136	0.397	0.002*∗∗*	0.385	0.002*∗∗*	0.364	0.004*∗∗*
Actually received institutional support	0.121	0.357	0.297	0.021*∗*	0.357	0.005*∗∗*	0.280	0.030*∗*
Actually received informative support	0.282	0.029*∗*	0.411	0.001*∗∗*	0.357	0.005*∗∗*	0.382	0.003*∗∗*
Satisfaction with actually received support	0.131	0.319	0.273	0.035*∗*	0.318	0.013*∗*	0.264	0.042*∗*
Protective buffering support	0.138	0.292	-0.016	0.903	-0.210	0.108	-0.018	0.894

## Data Availability

The data used to support the findings of this study are available from the corresponding author upon request.
